# Yeast metabolic chassis designs for diverse biotechnological products

**DOI:** 10.1038/srep29694

**Published:** 2016-07-19

**Authors:** Paula Jouhten, Tomasz Boruta, Sergej Andrejev, Filipa Pereira, Isabel Rocha, Kiran Raosaheb Patil

**Affiliations:** 1 European Molecular Biology Laboratory, EMBL, Heidelberg, Germany; 2 Department of Bioprocess Engineering, Lodz University of Technology, Poland; 3 University of Minho, Braga, Portugal

## Abstract

The diversity of industrially important molecules for which microbial production routes have been experimentally demonstrated is rapidly increasing. The development of economically viable producer cells is, however, lagging behind, as it requires substantial engineering of the host metabolism. A chassis strain suitable for production of a range of molecules is therefore highly sought after but remains elusive. Here, we propose a genome-scale metabolic modeling approach to design chassis strains of *Saccharomyces cerevisiae* – a widely used microbial cell factory. For a group of 29 products covering a broad range of biochemistry and applications, we identified modular metabolic engineering strategies for re-routing carbon flux towards the desired product. We find distinct product families with shared targets forming the basis for the corresponding chassis cells. The design strategies include overexpression targets that group products by similarity in precursor and cofactor requirements, as well as gene deletion strategies for growth-product coupling that lead to non-intuitive product groups. Our results reveal the extent and the nature of flux re-routing necessary for producing a diverse range of products in a widely used cell factory and provide blueprints for constructing pre-optimized chassis strains.

Baker’s yeast, *Saccharomyces cerevisiae*, is among the most widely used cell factories, utilized in biotechnological processes ranging from bioethanol to insulin production. A large knowledge base and considerable expertise exist for this fungus in both academia and industry, making it an attractive host for producing new products. Yet, the development of a new production-quality strain is a time-consuming as well as resource-consuming endeavor. Without substantial engineering of the host metabolism, yields from heterologous production pathways tend to remain low and economically unattractive^1^,^2^,^3^. Diverting the input carbon flux away from ethanol, biomass and CO_2_ production, and to the desired product, requires considerable expertise and several trial-and-error cycles of identifying and implementing genetic engineering targets. These hurdles hamper innovation and delay realization of final bioprocesses. Consequently, the idea of chassis cells, which are pre-engineered for the production of a variety of molecules, has received considerable traction in recent years^4^,^5^,^6^.

Since various compounds of biotechnological interest originate from a limited number of metabolic precursors, a major focus in the engineering of chassis strains has been on increasing the precursors’ availability. Primary precursors of recent interest include acetyl-CoA, malonyl-CoA, farnesyl diphosphate and aromatic amino acids^7^,^8^,^9^,^10^,^11^,^12^,^13^,^14^. Malonyl-CoA acts as a precursor for a range of products, including fatty acid ethyl esters and 3-hydroxypropionic acid. Furthermore, farnesyl diphosphate, which is derived from acetyl-CoA via the mevalonate pathway in native yeast metabolism, is a precursor for sterols and isoprenoids. Indeed, a strain with an improved farnesyl diphosphate pool has been suggested as a platform for products drawing precursors from the mevalonate pathway^14^. Aromatic amino acids and related metabolites are also precursors for many products, such as polyketides, vanillin and *p*-hydroxycinnamic acid.

In addition to increasing precursor supply, cellular redox balance has been another major target area to drive or enhance the production. Nissen *et al*.^15^ pioneered redox engineering in yeast by switching L-glutamate synthesis from an NADPH-dependent to an NADH-dependent reaction. This decreased anaerobic glycerol secretion and diverted more glucose to ethanol^15^. Further examples of successful redox engineering in *S. cerevisiae* include NADPH-consuming isoprenoid production^16^,^17^ and NADH-generating 2,3-butanediol production^18^.

The strain engineering strategies towards increasing precursor and cofactor availability can be rationalized using genome-scale metabolic models^19^,^20^. Such models are now available for a number of industrially relevant microbial hosts including *S. cerevisiae*. Furthermore, state-of-the-art computational methods allow rapid reconstruction of new models with as little information as genome sequence^21^,^22^. Genome-scale metabolic models form the basis of various algorithms for designing metabolic engineering strategies. These include OptKnock^23^ and its successor OptGene^24^, which identify gene deletion strategies to couple production flux to cell growth, where the main idea is that the cell’s evolution-driven biological objective ‘to grow’ would support the engineering objective of producing the compound of interest. Such growth-product coupling has been a success story in the field of model-guided rational design, with experimental demonstration for, among others, succinate^25^ and isoprenoid production^26^.

Biological objectives other than optimal growth may be relevant for designing metabolic engineering strategies. For example, endogenous flux regulation in non-evolved mutant strains may support production. Algorithms like MoMA^27^ and MiMBl^28^ have been shown to predict well the phenotypes of gene deletion mutants and can thus be used to identify metabolic engineering strategies. Moreover, methods have also been proposed to identify combinations of gene deletions and flux over-expressions to achieve high production levels^29^,^30^,^31^.

In this study, we address the design of a set of pre-optimized *S. cerevisiae* chassis cells. We aim at exploiting the modularity of cellular biochemistry from a production perspective, wherein different production pathways often share pre-cursors and co-factors. This, together with genome-scale metabolic models, enabled us to design modularized metabolic engineering strategies for re-routing carbon flux towards different groups of desired products.

## Methods

### Models for 29 products

**Figure 1:**
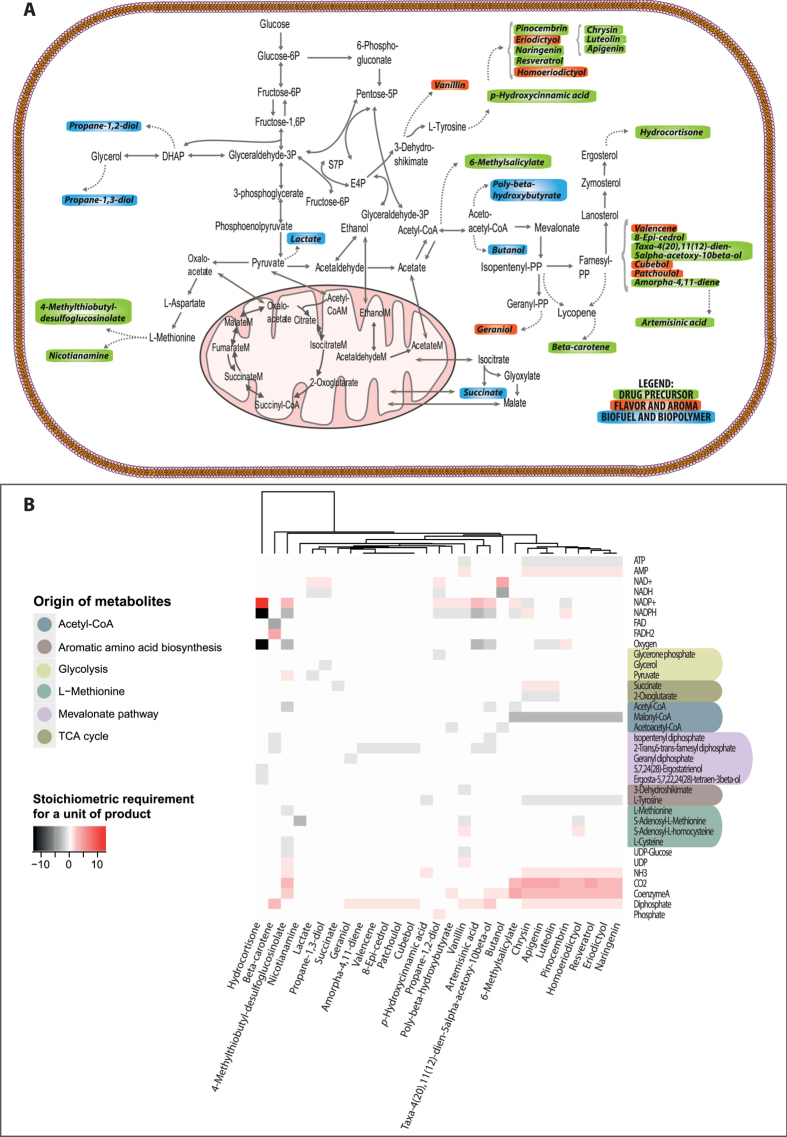
(**A**) The set of 29 heterologous and endogenous products considered in this study, overlaid on the native metabolic pathways of *S. cerevisiae*. Only the central metabolic pathways and metabolites relevant for these products are shown. The compounds are grouped according to their industrial use: drug precursors, flavor or aroma compounds, biofuels, and biopolymer precursors. (**B**) The heterologous pathways consume (black) and produce (red) compounds of the native metabolism. These pathways draw precursors as well as energy and redox cofactors from the native metabolism, in which the byproducts re-enter. Shown is the clustering of products according to these interactions with the yeast native metabolism.

We reconstructed biosynthetic pathways for 28 products requiring heterologous gene expression in *S. cerevisiae* based on literature data (Fig. 1, Supplementary information). In addition, succinic acid was used as a representative endogenous product. For simplicity and due to the unavailability of information, no additional energetic and precursor requirements were included for the synthesis of the heterologous enzymes or for the secretion of products or by-products. To represent the native metabolism of *S. cerevisiae,* we used the genome-scale metabolic model *i*FF708^32^. Among the available yeast models, *i*FF708 is particularly well suited for predicting metabolic flux phenotypes^33^. To further improve the model for metabolic engineering target identification, various curation and pre-processing steps were applied, using Matlab R2015a v. 8.5.0 with Cobra Toolbox v. 2.0.6^34^, as follows. Duplicated reactions were removed and the corresponding isoenzymes were included in the gene-reaction rules. Secretion of acetaldehyde and glucoseamine 6-phosphate were not allowed, as acetaldehyde is cytotoxic and is usually not secreted by yeast in large quantities^35^, and phosphorylated compounds can be assumed to remain intracellular^36^. To avoid growth-product couplings that are dependent on cytosolic proton balance, proton diffusion between the cytosol and extracellular medium was left unconstrained; indeed, the condition-dependent protonation states of metabolites and the effect on their transport across the cell membrane are not well known. The sterol biosynthesis pathway from lanosterol to zymosterol was revised to match the co-factor requirements as per the latest yeast genome-scale metabolic model (ref. 37 Yeast v. 7.6, yeast.sourceforge.net). Additionally, only the mitochondrial version of the L-alanine aminotransferase was considered^38^. Finally, the model was preprocessed to remove blocked reactions – reactions that cannot carry flux under minimal medium with glucose as the sole carbon source. The reduced model was then augmented with the required heterologous pathways to generate the corresponding 28 producer models.

### *In silico* metabolic engineering

To identify metabolic engineering targets, we used three distinct approaches: i) flux balance analysis (FBA)-based^39^ growth-product coupling; ii) MiMBl-based^27^ growth-product coupling and iii) flux variability analysis (FVA)^40^,^41^. The FBA-based approach was implemented as a variant of the OptKnock^22^ and RobustKnock^42^ algorithms. In brief, we calculated, for each of the simulated mutants, the minimal product secretion while constraining the growth to its maximum possible limit. To identify growth-product couplings using FBA, we simulated all combinations of single, double and triple reaction knockouts. Additionally, combinations of three reaction knockouts were simulated on the top of two pre-selected sets of manually chosen deletions. The two pre-selected sets were: i) *SDH3, SER3*, and *SER33* deletions, which have been shown to couple the glycine-serine pathway with growth^25^; and ii) *ICL1, KGD1,* and *PYC1* deletions, which we expected to reroute the flux around the TCA cycle, favoring the heterologous synthesis of apigenin, chrysin, and luteolin. From the obtained solutions, we selected those with nonzero growth yield (≥10^−2^ g biomass/mol glucose) and product yield above 90% of the maximum yield among all solutions. Solutions with product yield lower than 10^−3^ mol/mol glucose were not considered.

In the second approach, we used the MiMBl algorithm^28^ to predict the product flux for reaction knockout mutants. The use of the MiMBl algorithm requires a reference flux distribution (the “wild-type phenotype”) to calculate the metabolite turnover distance against. Since high-glucose conditions would be preferred for production, we generated a reference flux distribution representing a carbon catabolite repressed (CCR) metabolic state. For this, a parsimonious FBA (pFBA)^43^ simulation was performed using the following additional constraints: i) no respiration, and ii) the directionality of the glucose 6-phosphate isomerase reaction restricted towards the formation of fructose 6-phosphate. As the MiMBl algorithm has not yet been validated beyond double gene deletions, we restricted this approach to simulating single and double knockouts. The solutions were filtered to select those with growth yield ≥10^−3^ g biomass/mol glucose and product yield as in the case of FBA-based solutions.

Finally, reactions essential for the conversion of glucose into a particular product (at its maximum yield), and the minimal required flux through these, were identified using flux variability analysis^40^,^41^. The reactions thus identified constitute potential targets for gene overexpression.

All simulations were performed for minimal medium with glucose as the sole carbon source. To enumerate growth-product coupling solutions with the FBA and MiMBl algorithms, the IBM ILOG CPLEX v. 12.6.1 solver was accessed via C++ API. Flux variability analysis was performed in Matlab R2015a v. 8.5.0 using IBM ILOG CPLEX v. 12.6.1 function ‘cplexlp’. All further calculations to filter the growth-product coupling solutions and to identify the essential reactions from the flux variability data were done with Matlab R2015a v. 8.5.0.

### Cluster analysis

Heatmaps were generated using R v. 3.2.0^44^, with the function ‘heatmap.2’^45^. The clustering function used was ‘hclust’ with an average linkage method. The distance metrics ‘binary’ and ‘euclidean’ (accessed via function ‘dist’) were used for the product-essential flux data and stoichiometric precursor requirement data, respectively.

## Results

All 29 products considered here originate from only a handful of precursors in the endogenous metabolism of *S. cerevisiae* (Fig. 1A). Due to the requirements for cofactors and/or secondary precursors, the relationships between different products go beyond the sharing of primary precursors. Furthermore, the host cells need to either metabolize, or secrete, the byproducts of these heterologous pathways. Indeed, we observed product clusters (Fig. 1B) that are based on all three factors – main precursor, co-factor usage, and by-product formation. These clusters affirm the notion of modularity and set the stage for exploring chassis designs.

### Metabolic engineering targets for growth-product coupling

**Figure 2:**
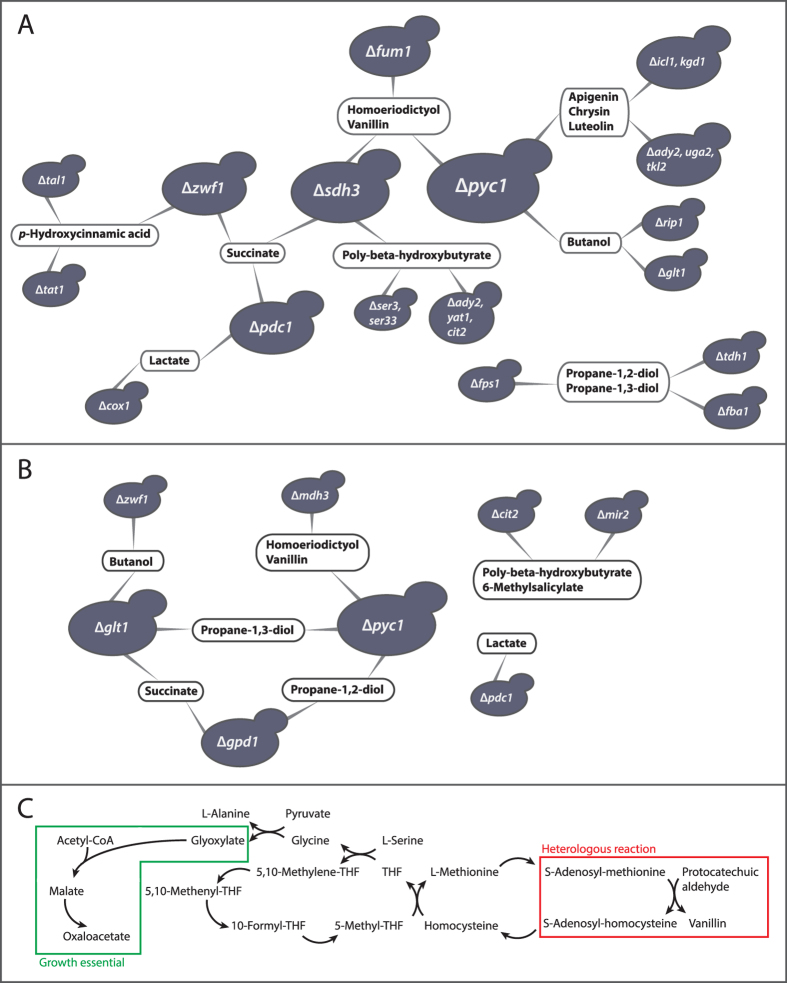
Examples of modularized growth-product coupling strain designs for *S. cerevisiae* identified with (**A**) the FBA-based algorithm (Methods), and (**B**) the MiMBl-based algorithm (Methods). These designs consist of reaction deletion targets, which are represented here in terms of the corresponding genes. In the case of isoenzymes, a representative gene, encoding the most active isoenzyme, is shown. (**C**) Illustration of how the production of vanillin becomes coupled to optimal growth through an L-methionine-Homocysteine interconversion in a deletion mutant where malate synthase and malate dehydrogenase reactions form an essential route to oxaloacetate.

We identified reaction deletion strategies that could couple the product flux to cell growth by applying an FBA-based approach to yeast metabolic models producing the 29 products considered here (see Methods). For all products, triple deletion strategies predicted higher product yields compared to single and double deletions (see Supplementary information). In the case of lactate production, some of the double deletion solutions were also considered among the potential strain designs as the corresponding predicted yields were above the set threshold (see Methods). We could identify growth-product coupling designs fulfilling our criteria for growth and product yields for 12 out of the 29 products (Fig. 2A). With the exception of products originating from the ergosterol or terpenoid pathway, growth-product coupling could be found for a broad range of products spanning precursors distributed throughout the native metabolic network of *S. cerevisiae*.

The potential for modular chassis designs is apparent in the solutions. In particular, we observed two major hub-deletions that are shared by several products: Δ*pyc1,2* (pyruvate carboxylase) and Δ*sdh3* (succinate dehydrogenase) (Fig. 2A). There are two pyruvate carboxylase isoenzymes (encoded by *PYC1, PYC2*) in *S. cerevisiae*. The mutant lacking both of the corresponding genes has been found to be inviable^46^, although the model predicts growth (i.e. putative false positive prediction). The strategy may still be implemented by deleting only one of the two genes or by down-regulating their expression. Knockout of the other predicted hub, succinate dehydrogenase (i.e. Sdh3_1, Sdh3_2), has been shown to disrupt the cyclic operation of the TCA cycle without affecting strain viability^25^.

### TCA cycle disruption forms a basis for multiple growth-product couplings

An example non-intuitive chassis design involves pyruvate carboxylase inhibition combined with the disruption of the TCA cycle at the succinate dehydrogenase (Sdh3_1 & Sdh3_2) reactions and fumarase (encoded by *FUM1*) nodes (Fig. 2A). In this mutant, the malate synthase from the glyoxylate cycle is the only remaining source of malate necessary for oxaloacetate synthesis. Malate synthase uses acetyl-CoA and glyoxylate as substrates. The latter is available, at optimal growth, only via the serine hydroxymethyl transferase (SHMT) route. This route produces glyoxylate from L-serine through glycine and glyoxylate aminotransferase (encoded by *AGX1*), while simultaneously converting homocysteine to L-methionine. The L-methionine derived SAM then couples SAM-consuming homoeriodictyol and vanillin production to growth (Fig. 2C).

In the growth-product coupling design for poly-beta-hydroxybutyrate production, the TCA cycle disruption at succinate dehydrogenase is instead combined with the blocking of the glycolytic L-serine synthesis. This renders the serine hydroxymethyl transferase (SHMT) route from glycine essential for L-serine synthesis. When glycine cannot be produced from L-serine, it can originate either from L-threonine aldolase, L-threonine dehydrogenase, or from transamination of glyoxylate. The growth-product coupling of poly-beta-hydroxybutyrate relies on L-threonine aldolase as a glycine source essential for optimal growth. This reaction produces acetaldehyde as a byproduct, which as a toxic metabolic intermediate, is readily converted to acetate and subsequently to acetyl-CoA. With further deletions of an acetate transporter (encoded by *ADY2*, in the model incorrectly annotated to *BPH1*), the carnitine shuttle of acetyl-CoA transport between compartments (e.g. Yat1), and the non-mitochondrial citrate synthase (i.e. Cit2), poly-beta-hydroxybutyrate production becomes coupled with optimal growth (Fig. 2). Assuming no mitochondrial acetyl-CoA synthetase activity (or additional disruption of any) when acetyl-CoA cannot enter the TCA cycle, poly-beta-hydroxybutyrate acts as a growth optimal sink for the overflow of acetyl-CoA.

The heterologous pathway for the synthesis of apigenin, chrysin, and luteolin includes a flavone synthase reaction utilizing alpha-ketoglutarate and succinate as a redox pair. Thus, a combination of a disruption of the TCA cycle at the alpha-ketoglutarate dehydrogenase node (Δ*kgd1*), of the glyoxylate cycle at the isocitrate lyase node (Δ*icl1*), and the inhibition of pyruvate carboxylase together offered a suitable background network for searching additional gene deletion strategies. In this background, we searched for strategies to couple the flavone synthase to growth. Indeed, a suitable design was identified which required blocking the conversion through succinate semialdehyde dehydrogenase (Δ*uga2*) in combination with the deletions of acetate transporter (Δ*ady2*) and transketolase (Δ*tkl2*) (Fig. 2A).

### *p*-hydroxycinnamic acid production forced by an imbalance in pentose phosphate pathway

*p*-hydroxycinnamic acid is synthesized from the aromatic amino acid L-tyrosine (Fig. 1). In the absence of aromatic amino acids in the growth medium, *S. cerevisiae* synthesizes these from the common precursors phosphoenolpyruvate and erythrose-4-phosphate. When the oxidative branch of the pentose phosphate pathway is blocked (i.e. Δ*zwf1/sol1,2,3,4/gnd1,2*) (Fig. 2A), transketolase provides growth essential pentose phosphates and erythrose-4-phosphate that are produced in one-to-one stoichiometry. However, this stoichiometry is not growth optimal but creates an overflow of erythrose-4-phosphate, which cannot be recycled in the absence of transaldolase activity (encoded by *TAL1*). When additionally L-tyrosine secretion is blocked (e.g. by deleting the corresponding transporter encoded by *TAT1*), *p*-hydroxycinnamic acid provides a growth optimal sink for the overflow of erythrose-4-phosphate.

### Production flux as an optimal route for recycling redox co-factors

In addition to the designs discussed above, which are based on carbon balancing, we observed a set of chassis designs that force a production pathway to be an optimal route for recycling redox co-factors. These designs (Fig. 2A) cover three products, lactate, butanol, and succinate. If respiration is blocked (e.g. Δ*cox1*) and pyruvate decarboxylase (encoded by *PDC1/5/6*) is inhibited, heterologous lactate dehydrogenase provides a growth-optimal NADH sink. Butanol synthesis is also NADH-consuming, making it a potential redox sink. When pyruvate carboxylase is inhibited and an increased NADH burden is created by rendering L-glutamate synthesis solely NADPH-dependent (i.e. Δ*glt1*), butanol synthesis becomes, under non-respiratory conditions (e.g. Δ*rip1*), a growth-optimal NADH sink.

Another example of coupling through redox balancing is the production of succinate – an intermediate metabolite of the TCA cycle. While a disruption of the TCA cycle at succinate dehydrogenase (encoded by *SDH3*) blocks the utilization of succinate, the inhibition of pyruvate decarboxylase (i.e. Δ*pdc1*) necessitates L-threonine aldolase to provide acetaldehyde to fulfill the requirement of cytosolic acetyl-CoA. The increased demand of flux through the L-threonine synthesis increases also the overall demand for NADPH for growth. This increase is due to the NADPH-requiring aspartic beta-semialdehyde dehydrogenase. Additionally, blocking the flux through the oxidative branch of the pentose phosphate pathway (i.e. Δ*zwf1/sol1,2,3,4/gnd1,2*), the main source of cytosolic NADPH, puts the alternative NADPH sources under pressure to increase flux. While isocitrate dehydrogenase (i.e. the isoenzyme encoded by *IDP2*) provides NADPH, succinate semialdehyde dehydrogenase (i.e. in the GABA shunt bypass of the TCA cycle) also produces NADPH and succinate. Succinate production thus becomes coupled to optimal growth as an optimal route for NADPH formation.

### Disruption of glycolysis for growth coupling of glycerol pathway products

Propane-1,2-diol originates from dihydroxyacetone phosphate and propane-1,3-diol from glycerol (Fig. 1). A suggested strategy for the growth coupling of these products is to knock out the glycerol transporter (i.e. Δ*fps1*) while disrupting glycolysis at the glyceraldehyde 3-phosphate dehydrogenase (encoded by *TDH1/2/3*) and the fructose bisphosphate aldolase (encoded by *FBA1*) nodes (Fig. 2A). In this network disrupted in glycolysis, *de novo* NAD synthesis through kynurenine pathway and L-tryptophan synthesis would act as the sources of essential pyruvate, maintaining cell viability. The deletions would also result in flux reorganization where, for optimal growth, glyceraldehyde 3-phosphate from the pentose phosphate pathway would be directed to dihydroxyacetone phosphate and further to the two products.

### Metabolic engineering targets for regulatory growth-product coupling

The chassis designs discussed above assume growth optimality of the engineered strains. This would require, in most cases, a long-term adaptive evolution leading to an extensive rewiring of the regulatory networks. As an alternative approach, we used the MiMBl algorithm^28^, which predicts the fluxes in engineered strains without assuming growth optimality, but assumes minimal changes to the flux regulation in the reference strain (see Methods). In the strain designs identified using MiMBl, the endogenous flux regulation is expected to force flux through the production pathway so as to minimize the changes in metabolite turnovers in comparison to the wild type strain. In this study, as a reference flux distribution we considered glucose-repressed growth without oxidative phosphorylation. We identified such regulatory growth-product coupling strategies for 9 products (Fig. 2B), including 6-methylsalicylate for which no solutions were identified using the FBA-based algorithm. Interestingly, many deletion targets, including pyruvate carboxylase and pyruvate decarboxylase, overlap with the FBA-based designs, marking these as attractive engineering strategies.

### Gene overexpression design strategies

The growth-product coupling strategies described above did not cover all of the 29 products. Moreover, the yields in such designs are inherently limited due to stringent growth requirements built in to the designs. This limitation could potentially be overcome by increasing the number of deletions, which may become impractical from an experimental perspective. Furthermore, increased deletions may not necessarily provide high yield solutions for all products^24^. In contrast, algorithms based on flux overexpression have been shown to suggest potentially high yield strategies in most cases^29^. We used flux variability analysis (FVA) to identify flux overexpression targets and the corresponding modular chassis designs spanning many products.

**Figure 3:**
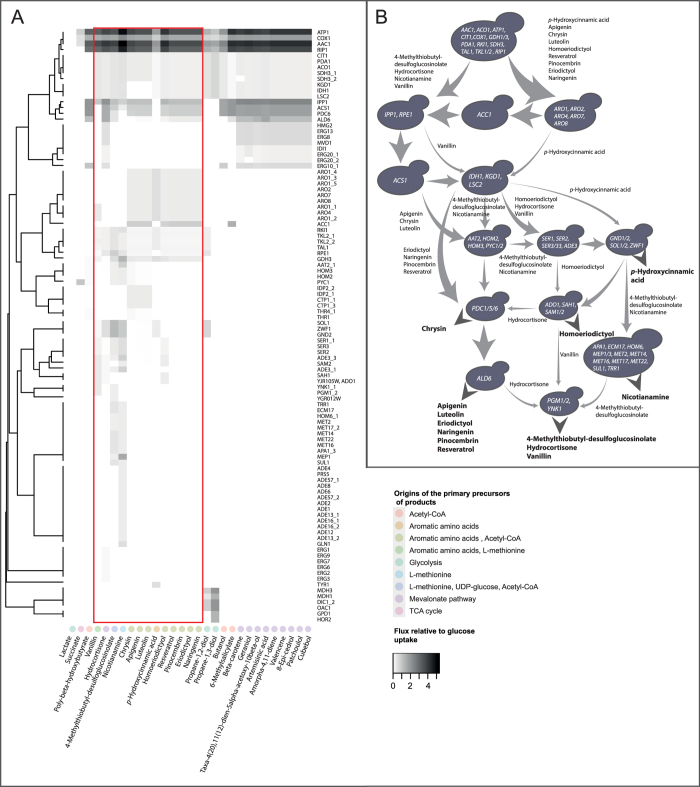
Modular chassis designs based on the shared product-essential reactions (overexpression targets) for optimal conversion of glucose into the selected products. (**A**) Clustering of product-essential reactions based on their utilization across different products. Only the reactions shared by sub-groups of products are shown while e.g. glucose transport and phosphorylation reactions shared by all are not included in the figure. The main origins of the products in the native metabolism are shown with color codes. (**B**) An example modular chassis design shown as a flow chart of module utilization by the products highlighted in the heatmap in (**A**).

For each product, we identified reactions essential for obtaining optimal yield from glucose and also recorded the minimal required fluxes through these. Different product groups share many essential reactions among them, forming the basis for modular chassis designs (Fig. 3A,B). The required flux through these reactions, however, might differ from product to product. From an experimental implementation perspective, this could be tackled by using a library of promoters with different relative activities. The identified designs provide a blueprint for gene overexpression strategies that can be applied to multiple products.

## Discussion

In this study, we provide modularized chassis designs for producing 29 different compounds of industrial interest in the widely used cell factory *S. cerevisiae*. The designs consist of both gene deletion targets for growth-product coupling as well as gene overexpression targets for boosting product yield. The growth-coupling algorithms used in this study have already been used to improve production of succinate, vanillin, and sesquiterpenes^25^,^26^. The vanillin- and isoprenoid-producing strains share a common strategy of generating NADPH surplus to be re-routed to the product pathway. Indeed, this shared basis of production improvement is consistent with the here-proposed concept of modular chassis design.

In addition to the specific targets for metabolic engineering, the designs identified here provide general insights into the flux re-routing associated with heterologous production. Our results highlight growth coupling as a fundamental strategy that can be applied to diverse products. Growth-product coupling arises when the product flux becomes either an unavoidable or an optimal byproduct of growth. In the case of an unavoidable byproduct, the optimal growth route includes a cleavage reaction generating an essential precursor for growth and a precursor for the product. In the optimal-byproduct case, production becomes an optimal route for redox/cofactor balance. The mechanisms underlying such dependencies between growth and product flux are sensitive to the overall stoichiometry of the product formation and are not necessarily dictated by the main precursor. Consequently, many products grouped according to the shared targets in a non-intuitive fashion. This non-intuitive clustering is also observed in the case of MiMBl-based regulatory growth-product coupling as well as in the case of overexpression targets (based on flux variability analysis). Together, the chassis design strategies underline the need and usefulness of a comprehensive simulation approach as presented here. The proposed chassis designs at present do not directly consider kinetic or regulatory constraints on flux distributions. Experimentally measured metabolic phenotypes of a subset of the suggested mutant strains could provide a basis for further refinement of the chassis designs, possibly including modifications of signaling and regulatory pathways.

The modularized chassis designs proposed here offer an excellent starting point for experimental validation. The proposed deletion strains can be engineered using the broad gene disruption toolbox available for yeast, including tools for simultaneous introduction of multiple gene deletions^48^,^49^,^50^. On the pathway front, new tools enable large pathways to be assembled and integrated in a high-throughput fashion^51^,^52^,^53^. These can also be used to integrate large pathways as bio-bricks into different strains. Thus, the bottleneck of metabolic engineering is shifting from pathway assembly to the optimization of the host metabolism. Given the large size of the native metabolism, the space of possible network modifications is extremely large and infeasible to explore in an exhaustive manner. Thus, the chassis designs and methods proposed here offer an excellent starting point to tackle this challenge towards developing industrially attractive cell factories. In particular, the modular design enable using a pre-optimized strain for a family of products, and thus reducing the strain development time.

## Additional Information

**How to cite this article**: Jouhten, P. *et al*. Yeast metabolic chassis designs for diverse biotechnological products. *Sci. Rep.* **6**, 29694; doi: 10.1038/srep29694 (2016).

## Supplementary Material

Supplementary Information
